# Primary Hyperparathyroidism in Older People: Surgical Treatment with Minimally Invasive Approaches and Outcome

**DOI:** 10.1155/2012/539542

**Published:** 2012-06-12

**Authors:** Chiara Dobrinja, Marta Silvestri, Nicolò de Manzini

**Affiliations:** Division of General Surgery, Department of Medical and Surgical Sciences, Hospital of Cattinara, University of Trieste, 34149 Trieste, Italy

## Abstract

*Introduction*. Elderly patients with primary hyperparathyroidism (pHPT) are often not referred to surgery because of their associated comorbidities that may increase surgical risk. The aim of the study was to review indications and results of minimally invasive approach parathyroidectomy in elderly patients to evaluate its impact on outcome. *Materials and Methods*. All patients of 70 years of age or older undergoing minimally approach parathyroidectomy at our Department from May 2005 to May 2011 were reviewed. Data collected included patients demographic information, biochemical pathology, time elapsed from pHPT diagnosis to surgical intervention, operative findings, complications, and results of postoperative biochemical studies. *Results and Discussion*. 37 patients were analysed. The average length of stay was 2.8 days. 11 patients were discharged within 24 hours after their operation. Morbidity included 6 transient symptomatic postoperative hypocalcemias while one patient developed a transient laryngeal nerve palsy. Time elapsed from pHPT diagnosis to first surgical visit evidences that the elderly patients were referred after their disease had progressed. *Conclusions*. Our data show that minimally invasive approach to parathyroid surgery seems to be safe and curative also in elderly patients with few associated risks because of combination of modern preoperative imaging, advances in surgical technique, and advances in anesthesia care.

## 1. Introduction

Primary hyperparathyroidism (pHPT) is one of the most common endocrine diseases with prevalence rates of about 3 per 1,000 in the general population and up to 1 per 100 in the elderly. The chances of developing pHPT increase with age, and patients are most often diagnosed in the sixth and seventh decades of life [1].

Changes in endocrine systems, including loss of skeletal mass, are common or certain in older persons. Skeletal mass increases in most individuals until about the age of 20. It remains stable until about the age of 35 and thereafter declines at a relatively steady rate throughout the remainder of life. Decreased skeletal mass associated with increasing age is the result of a series of changes associated with aging: calcium absorption/transport in the intestinal mucosa and mineral metabolism [2], vitamin-D-dependent calcium absorption, and calcium intake generally decreasing with age [3]. As renal function decreases with age, parathyroid hormone (PTH) increases with augmented osteoclastic and osteoblastic activity [4–7]. Although, after the introduction of serum calcium determination in the routine biochemical screening in the early 1970s, most patients referred to surgery are asymptomatic, clinical manifestations of pHPT, including disabling fractures, are frequent in the elderly [8].

Elderly patients with pHPT are often not referred for surgical intervention because of their associated comorbidities that may increase surgical risk.

For the reason that pHPT is more common in the elderly [1] and because minimally invasive techniques in parathyroid surgery have recently been demonstrated safe with improved perioperative outcome [9–14], the aim of our study was to review indications and results of minimally invasive parathyroidectomy in patients 70 years of age and older (elderly) to evaluate the safety and efficacy of outpatient minimally invasive parathyroidectomy (MIP) and minimally invasive video-assisted parathyroidectomy (MIVAP).

## 2. Materials and Methods

A review was conducted of a prospectively collected database of all patients undergoing parathyroidectomy at the Department of General Surgery of University of Trieste. During the last 5 years, from May 2005 to May 2011, 83 patients with pHPT underwent parathyroidectomy. 37 (44.58%) patients of 70 years of age or older were reviewed. MIVAP by anterior approach, technique previously described [13, 15], was proposed for patients with sporadic pHPT due to a single gland disease, an adenoma smaller than 35 mm as demonstrated by preoperative imaging, unequivocally preoperative localization by ultrasonography and 99mTc-SestaMIBI scanning, no associated giant goiter, no suspected carcinoma of the thyroid, no secondary or recurrent hyperparathyroidism, no previous neck surgery, and no previous radiation to the neck. We used the operative technique first described by Miccoli et al. in 1997 and 1998 [16, 17], without carbon dioxide insufflation. The procedure, in which four surgeons are involved (first operator, one surgeon assisting the first operator, one surgeon holding retractors, and one surgeon holding the endoscope), was performed through a single 20 mm skin incision in the central neck, 1-2 cm above the sternal notch. The patient, under general anaesthesia, is placed in a supine position; the neck is in slight extension. Dissection was performed under endoscopic vision, using small conventional retractors and needlescopic (2 mm) reusable instruments. Video assistance was obtained using a 30-degree 5 mm endoscope. The thyroid lobe was retracted medially and the adenoma was extracted after clipping its pedicle.

MIP, defined as open “directed” parathyroidectomy (unilateral targeted cervical exploration) with a minimally invasive approach (smaller cervical incision and less cervical dissection than traditional open technique necessitating bilateral cervical exploration), was proposed for patients with sporadic pHPT due to a single gland disease, preoperative localization of one enlarged parathyroid gland on ultrasonography or on 99mTc-SestaMIBI scanning, no associated suspected thyroid malignancies, and no secondary or recurrent hyperparathyroidism.

MIP was proposed when preoperative ultrasonography did not localize certainly the adenoma or when there were benign thyroid nodules associated with pHPT.

Technically, MIP is the open focused procedure that is performed without the videoscope and requires greater neck extension, a 3 to 5 cm incision, and larger subplatysmal flaps than MIVAP. In all other aspects, the procedure is practically identical.

Intraoperatively, a quick parathyroid assay was used to measure intact parathyroid hormone levels during the last 25 surgical procedures (PTHIO): before surgery and then 5, 10, and 15 minutes after excision of the adenoma, peripheral blood was drawn.

Intact PTH assay, both in routine and intraoperative mode, was performed on samples collected into potassium EDTA anticoagulant tubes according to the routine and intraoperative procedure of the Access Immunoassay System Intact PTH, respectively, a paramagnetic particles, chemiluminescent immunoassay for the quantitative determination of PTH levels in human serum and plasma on Access2 Beckman Coulter (Fullerton).

The calibration, valid up to 28 days, was performed for routine and intraoperative modes using separate calibration cards and two control levels, low and high, were double assayed before and after surgical operation. The assay imprecision was evaluated by testing 2 levels of controls generating a total of 20 assay, 2 replicate per assay, over 10 days [18].

The analytical range for PTH and PTHIO assay was 1–3500 pg/mL, and 6–3500 pg/mL respectively; the reference interval for PTH values was 11–73 pg/mL.

A decrease in iPTH levels of more than 50% in the 5-minute postexcision sample below the preincision value, as suggested by Irvin et al. [19, 20], was the criterion used to indicate that the offending gland has been excised and the remaining parathyroids glands were not hyperfunctioning.

In summary, the surgical procedures were considered successful when more than a 50% decrease in preexcision PTH levels was observed after 5 minutes.

Preoperative informed consent was obtained from all patients [15].

pHPT was biochemically confirmed before surgery, and patients were selected for parathyroidectomy based on symptoms and according to guidelines for surgical intervention in asymptomatic pHPT. Preoperative planning included preoperative calcemia (normal range: 8.50–10.50 mg/dL), and PTH levels (normal range: 11–73 pg/mL). All patients underwent preoperative investigations of vocal cord function.

Data collected included patients' demographic information, biochemical pathologic, time elapsed from pHPT diagnosis to surgical intervention, PTHIO measures operative findings, complications, and results of postoperative biochemical studies.

### 2.1. Statistical Analysis

All patients of 70 years of age or older, undergoing parathyroidectomy at our department from May 2005 to May 2011, were included in a database. In an Excel spreadsheet were entered data of patients, surgical procedures, length of surgical procedures, length of stay, biochemical analysis, cytology, histology, PTHIO measures, complications, and results of postoperative biochemical studies.

Statistical analysis was performed using the “R 2.13.1” software (http://www.rproject.org/).

Continuous data were expressed as the mean or median ± standard deviation, as appropriate, while discrete data as a finite value or percentage.

**Figure 1:**
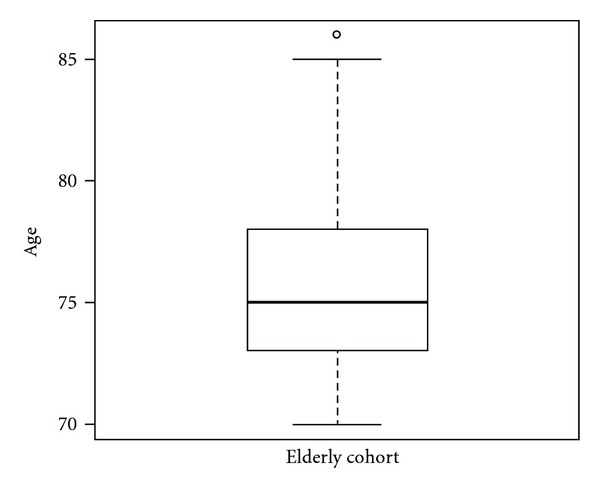
Box plot regarding age. It represents the distribution by age of pHPT in our elderly cohort made up of 37 patients during the last 5 years of activity of the Department of General Surgery of University of Trieste. Elderly mean age at time of operation was 76.08 years (range: 70–86 years). The median age was 75 years and corresponds to the dark thickened band near the middle in the box plot, drawn vertically. The box in the figure shows the lower and upper quartiles of age (range 73–78 years) while whiskers show values below the 25th (from 70 to 72 years) and above the 75th (from 79 to 85 years). The upper dot not included between the whiskers means a patient 86 years old at time of operation.

**Figure 2:**
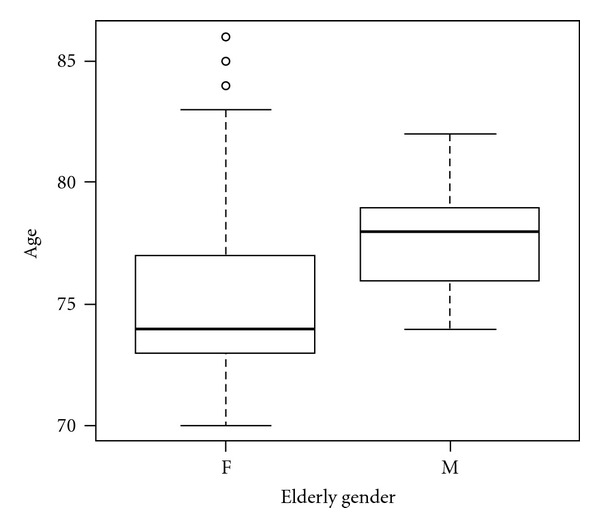
Box plot regarding age and sex. The figure shows the distribution by age, represented vertically, and gender, horizontally, of pHPT in our elderly cohort The box plot highlights that, over 5 years of activity of our Department, there was a major incidence of pHPT in females than males (28 versus 9) and that the age of onset of disease was lower in elderly females than elderly males. In fact, for females, the median age was 74 years and lower and upper-quartiles of age ranged from 73 to 77 years; 3 outlier patients 84, 85, and 86 years old at time of operation. On the other side, for males: the median age was 78 years and lower and upper quartiles of age ranged from 76 to 79 years.

The population's ages were forced with two box plots display (Figures 1 and 2) to show graphically the distribution of elderly population.  Figure 1 shows all elderly patients in relation to age while Figure 2 represents the differences between elderly males and elderly females regarding age.

The Fisher exact test was employed to correlate the complications between the two techniques.

A *P* value less than 0.05 was considered statistically significant.

## 3. Results and Discussion

During the last 5 years, from May 2005 to May 2011, 37 patients, 28 women and 9 men, of 70 years of age or older were analysed. Mean age at time of operation was 76.08 ± 4.2 (range: 70–86 years), and median was 75 (Figure 1). For females, mean age was 75.68 ± 4.5 (range: 70–86 years), and median 74; for males, respectively, 77.33 ± 2.6 (range: 74–82 years) and  78 ± 2.6  (Figure 2).

Mean time elapsed from pHPT diagnosis to surgical intervention was 9 months ranging from 2 to 36 months.

Mean preoperative serum calcium level was 11.43 mg/dL (range: 10.3–15 mg/dL). Among the 37 elderly patients who had parathyroidectomy, 11 (29.73%) had concomitant thyroid surgery. The mean overall PTHIO drop was a drop of 18.38% in T1 and 59.91% in T2.

Totally, 29 MIP and 8 MIVAP were performed.

Mean operative time was 66 minutes (range: 32–100 minutes) for MIP and 96 for MIVAP (range: 40–148), respectively. In all patients final histology showed benign disease. Surgical cure of pHPT was achieved in all patients with serum calcium levels normalization.

The average length of stay was 2.8 days (range: 1–13 days). 11 patients (29.73%) were discharged within 24 hours after their operation.

Overall morbidity was 18.91% (7 patients), and precisely postoperative complications included 6 (16.21%) transient symptomatic postoperative hypocalcemias (complete recovery after 13 days) while one patient developed transient laryngeal nerve palsy (2.70%).

No definitive laryngeal nerve palsies, no definitive hypocalcemias (lasting more than 6 months after surgery), no persistent pHPT, and no recurrent pHPT were observed.

Postoperative complications in MIVAP group included 1 (12.5%) transient hypocalcemia, whereas, in the MIP group, postoperative complications included 5 (17.24%) transient hypocalcemias (*P* = 1.458) and 1 (3.44%) temporary laryngeal nerve palsy (complete recovery after 1 month) (*P* = 0.8947).

At a mean follow-up of 31 months ranging from 6 to 66 months, all patients are normocalcemic. 

Primary hyperparathyroidism is a common endocrine disorder in the elderly. After the introduction of routine serum calcium measurements in the 1970s, facilitating the diagnosis of asymptomatic pHPT, epidemiological studies from the United States and Europe reported a higher prevalence than previously presumed [1, 21].

In a literature review, in accordance with an age-dependent increase in pHPT, the population- adjusted incidence of parathyroidectomy resulted in being higher in persons aged ≥50 years. The peak of pHPT incidence was observed in patients aged 70–74 years (12.7/100,000), with a decline over 75 [21, 22].

Parathormone secretion tends to increase slightly with age. The relation can be attributed to decreased calcium and vitamin D intake (and possibly decreased sun exposure) and to kidney failure as causes a reduction of 1,25hydrosilation of vitamin D.

Bone mass, influenced by the effect of PTH, declines gradually with age; the decline accelerates after menopause in women and continues indefinitely. This loss of bone contributes to an increased risk of fracture and increased probability of falls.

Since the introduction of routine calcium screening and multichannel biochemical testing, the majority of pHPT patients are diagnosed at an early stage and pHPT patients are commonly referred to as “asymptomatic.” Guidelines for managing asymptomatic pHPT have been outlined using objective indications for surgical intervention [23]. Surgical removal of abnormal parathyroid tissue is the only curative treatment for pHPT.

Despite a continuing increase in pHPT hospitalisation rates with older age, elderly patients are often not referred for surgical intervention [24]. Several factors could affect the decision of surgery: the age, the number of comorbidities, and the remaining life expectancy.

Time elapsed from pHPT diagnosis to first surgical visit evidences that the elderly patients in our series were referred after their disease had progressed, with advanced bone disease, and very high PTH and calcium serum levels.

Although encouraging outcomes of surgical treatment in elderly patients with pHPT have been reported [9–14] there may exist a reluctance to offer the surgical intervention in particular from endocrinologists; moreover, elderly patients often fear parathyroidectomy. In fact, the reason of this delay is not only ascribed to physician or surgeon. It is necessary to take in account the psychological aspect of elderly patients who often admit their panic versus surgical intervention and then their refusa. Elderly patients as a result are often not allowed to improve in quality of life, bone health, neuromuscular function, psychiatric symptoms, and the decrease in morbidity and mortality that are associated with cure of pHPT [25–27].

Although medical therapy exists for the decreased bone mass caused by pHPT, this therapy based on Calcium Sensor Mimetics is often not tolerated while surgical therapy is definitive and offer better results.

In the past surgeons and physicians have been less inclined to operate on or to refer to surgery elderly pHPT patients because of the risks associated with standard cervical exploration, general anesthesia, endotracheal intubation, long hospital stay, and postoperative complications.

But it is necessary to consider that although, historically, pHPT has been treated by standard cervical bilateral (four glands) exploration, single adenomas are responsible for pHPT in >80% of patients and resection of the gland involved is curative [28, 29].

In recent years, improved preoperative localization techniques and the availability of intraoperative parathyroid hormone monitoring have opened the way for minimally invasive procedures, and several procedures have been described [16, 17, 30–32]. Previous studies have shown that parathyroidectomy today can be performed as safely in elderly patients as in their younger counterparts [14, 33] with low complication rate also in elderly patients and with discharge after an overnight stay.

In our series all patients underwent general anesthesia, but in the literature are described also minimally invasive parathyroidectomy for primary hyperparathyroidism, under light sedation, or using locoregional cervical block anesthesia [34–36]. These new advances in anesthesia care reflect the possibility to operate on also patients who are not eligible for general anesthesia, for example, for serious associated cardiovascular comorbidities.

In this study, we present our results of elderly patients who underwent parathyroidectomy by minimally invasive approach. About 30% of patients were discharged within 24 hours after their operation. Mean hospital stay was about 2-3 days, data similar with other results reported for younger patients [10, 11, 14]. Also the morbidity in this series is not dissimilar to other reports [14, 16]. Anyway, it is indispensable to consider, as reported by Thomas et al. [37], that elderly patients sustain more morbidity following parathyroidectomy related to their comorbidities such hypertension and coronary and pulmonary diseases than those related to surgical procedure. Moreover, older individuals often live alone and early discharge is very difficult. In our series, one patient had a very long hospital stay (13 days). There was a 76-year-old woman who underwent minimally invasive parathyroidectomy and total thyroidectomy associated, in which symptomatic postoperative hypoparathyroidism occurred and, in spite of substitutive therapy, serum calcium levels resulted at all controls in being lower than normal values. Furthermore, the patient was not autosufficient with various disabilities and asked to remain in hospital until complete normalization of serum calcium levels.

## 4. Conclusions

Our data show that minimally invasive approach to parathyroid surgery, either video assisted or with only open smaller neck incision and unilateral cervical exploration, seems to be safe and curative also in elderly patients. Furthermore, risks associated to this kind of surgery are nowadays considerably lower because of the combination of modern preoperative imaging, advances in surgical techniques and advances in anesthesia care. In view of this we recommend early surgical referral for pHPT in all patients regardless of age. Further research, with a greater number of patients is necessary to identify a precise benefit-risk assessment.
